# Metabolic derangements and reduced survival of bile-extracted Asiatic black bears (*Ursus thibetanus*)

**DOI:** 10.1186/s12917-019-2006-6

**Published:** 2019-07-29

**Authors:** Monica Kaho Herkules Bando, O. Lynne Nelson, Clark Kogan, Rance Sellon, Michelle Wiest, Heather J. Bacon, Mandala Hunter-Ishikawa, Wendy Leadbeater, Koji Yamazaki, Yipeng Jin, Takeshi Komatsu, David McGeachy

**Affiliations:** 1 Veterinary Clinical Sciences, College of Veterinary Medicine, Washington State University, P.O. Box 646610, 100 Grimes Way, ADBF, Pullman, Washington 99164-6610 USA; 2 Center for Interdisciplinary Statistical Education and Research (CISER), Washington State University, Abelson Suite 227, Office 221, Pullman, Washington 99164 USA; 3 Department of Statistical Science, University of Idaho, 875 Perimeter Drive, MS 1104, Moscow, ID 83844-1104 USA; 4 Jeanne Marchig International Centre for Animal Welfare Education, The Royal (Dick) School of Veterinary Studies, University of Edinburgh, Easter Bush Veterinary Centre, Roslin, Midlothian, EH25 9RG Scotland; 5 Ensessa Kotteh Wildlife Rescue, Born Free Foundation Ethiopia, PO Box 3138/1250, Addis Ababa, Ethiopia; 6 Veterinary Specialty Hospital, Lucky Centre, 1/F, 165-171 Rd, Wan Chai Road, Wan Chai, Hong Kong; 7 Forest Ecology Laboratory, Department of Forest Science, Tokyo University of Agriculture, 1-1-1 Sakuragaoka, Setagaya-ku, Tokyo 156-8502 Japan; 8 Clinical Department, College of Veterinary Medicine, China Agricultural University, Haidian District, Yuanmingyuan Xi Lu #2, Beijing, 100193 People’s Republic of China; 9 Kumakuma-en Kitaakita, 1-39 Ani-utto-Jinba, Kitaakita, Akita 018-4733 Japan; 10 Wildlife Research Division, Environment and Climate Change Canada, CW405, Biological Sciences, University of Alberta, Edmonton, AB T6G 2E9 Canada

**Keywords:** Asiatic black bear, Bear bile farming, Bile-extraction, Biochemistry, Hematology, Survival, *Ursus thibetanus*

## Abstract

**Background:**

Across China and Southeast Asia, an estimated 17,000 bears are currently farmed for bile, primarily for traditional medicines. Depending on country, bile is extracted daily via transabdominal gallbladder fistulas, indwelling catheters, or needle aspiration. Despite claims that bears do not develop adverse effects from bile extraction, health issues identified in bears removed from bile farms include bile-extraction site infections, abdominal hernias, peritonitis, cholecystitis, hepatic neoplasia, cardiac disease, skeletal abnormalities, and abnormal behaviors. We present a comprehensive assessment of the effects of bile farming by comparing serum biochemical and hematological values of bears from farms that were bile-extracted (BE) and bears from farms not bile-extracted (FNE) with bears from non-farm captive (ZOO) and free-range (FR) environments. We hypothesized BE bears would have significant laboratory abnormalities compared to all non-extracted bear groups. We also hypothesized BE bears would have reduced long-term survival compared to FNE bears despite removal from farms.

**Results:**

BE bears exhibited the highest values and greatest variation (on a population level) in laboratory parameters compared to all non-extracted bear groups particularly for alanine transaminase, gamma glutamyltransferase (GGT), total bilirubin (TBIL), alkaline phosphatase (ALKP), blood urea nitrogen, creatinine (CREA), and total white blood cell count. Significant differences were detected between bear groups when accounting for season, sex, and/or age. BE bears exhibited greater mean serum GGT compared to all non-extracted bear groups, and the odds of having elevated TBIL were 7.3 times greater for BE bears, consistent with hepatobiliary disease. Biochemical parameter elevations in BE bears persisted up to 14 years post-rescue, consistent with long-term effects of bile-extraction. BE bears that arrived with elevated CREA and ALKP had median survival times of 1 and 4 years respectively, and regardless of laboratory abnormalities, BE bears had significantly shorter survival times compared to FNE bears.

**Conclusions:**

Our results provide strong evidence that bile extraction practices not only represent a temporary constraint for bears’ welfare, but confer distinct long-term adverse health consequences. Routine laboratory panels may be insensitive to detect the extent of underlying illness in BE bears as these bears have significantly reduced survival regardless of biochemical assessment compared to FNE bears.

**Electronic supplementary material:**

The online version of this article (10.1186/s12917-019-2006-6) contains supplementary material, which is available to authorized users.

## Background

Bear bile has been used for centuries in traditional medicines to treat conditions ranging from fever and liver disease, to hemorrhoids and epilepsy [1]. Despite the availability of alternatives to bear bile [2, 3] up to 17,000 bears are estimated to be currently farmed for bile (legally and illegally) across China, South Korea, Vietnam, Laos, and Myanmar [4, 5]. The predominant species farmed for bile is the Asiatic black bear (*Ursus thibetanus*), along with Eurasian brown bears (*Ursus arctos arctos*), and Malayan sun bears (*Helarctos malayanus*).

Bile farming is controversial as it involves daily bile extraction over many years from living bears through surgically created transabdominal gallbladder fistulas or indwelling catheters (in China) [1, 4, 6, 7]. Husbandry conditions on bear bile farms include small cage confinement (severely restricting mobility), poor hygiene, inappropriate nutrition, and lack of provision of veterinary care [6–8]. Numerous health issues have been identified in bears removed from bile farms including, but not limited to, emaciation, bile-extraction site infections, abdominal hernias, cholecystitis, gallbladder polyps, cholelithiasis, abdominal abscesses, septicemia, and peritonitis [6, 8]. Additional clinical findings include abnormal behaviors and severe stereotypy, fractured teeth, spondylosis and degenerative joint disease, and missing digits, paws, or limbs presumably from snare traps [6]. Leading causes of death in previously bile-farmed bears are hepatobiliary neoplasia, hindlimb paralysis and deteriorating mobility, and cardiovascular disease, including congestive heart failure and aortic aneurysm rupture/dissection [6, 9]. Bile farmed bears are thought to have a greater prevalence and severity of disease, including comorbidities, than expected for most captive bears, and the types of disease, such as ruptured aortic aneurysm, appear unusual for bear species in general [9, 10].

Due to public concerns, regulatory changes drafted in 1996 were meant to address and rectify health and welfare issues of bears on bile farms [6, 11]. Subsequently, statements have been made that bears on bile farms are healthy and bears undergoing bile extraction do not experience adverse long term effects [12]; however, evidence to support this claim is lacking. In addition, access to bile farms has become increasingly restricted and information regarding husbandry, management, morbidity, and mortality of bears on bile farms is limited or unavailable [6, 8].

Based on reports of compromised health of bears from bile farms, we suspected bears that experience chronic bile extraction were more likely to have systemic illness than bears that do not undergo bile extraction. Specifically, we hypothesized that bile-extracted (BE) bears would show significant differences in biochemical and hematological variables, particularly related to the hepatobiliary and renal systems, when compared to non-extracted bears. We also hypothesized that BE bears would exhibit reduced long-term survival compared to non-extracted bears. We assessed the effect of bile farming and bile extraction on bears in two ways. First, we compared serum biochemical and hematological variables between bears arriving from bile farms, both bile-extracted (BE) and farm-not-extracted (FNE), with captive bears housed in presumably healthier conditions (ZOO), and free-range (FR) bears. Secondly, amongst bears from bile farms exposed to similar husbandry conditions, we compared survival of bears exposed to bile extraction with bears not exposed to bile extraction.

To the authors’ knowledge, there has yet to be a description of routine laboratory parameters of bile-extracted bears to assess the effects on health and survival of bile farming and bile extraction on bears. Serum biochemical and hematological parameters are recognized diagnostic tools in the assessment of individual and population health, physiology, as well as diagnosis and monitoring of progression or resolution of disease in domestic and wildlife species. Using these routine diagnostics, we hope to elucidate the health status of bile-farmed bears and determine whether bile-extraction impacts the long-term health and survival of bears. Findings from this study may also prompt additional biomarkers for early detection of disease and improve the long-term management of bears rescued from bile farms.

## Results

### Serum biochemical and hematological parameter comparisons between bear groups (raw data)

The BE group exhibited the highest individual values and the widest range of values for serum biochemical analytes (except albumin) and hematological parameters. This observation was particularly true for alanine transaminase (ALT), gamma-glutamyl transferase (GGT), alkaline phosphatase (ALKP), total bilirubin (TBIL), blood urea nitrogen (BUN), creatinine (CREA), and total white blood cell count (TWBC). Means, medians, ranges, and standard deviations of eleven serum biochemical analytes and two hematological parameters of interest from BE, FNE, ZOO, and FR bears are presented in Tables 1 and 2.

**Table 1:** Comparison of serum enzyme and metabolite values across four groups of Asiatic black bears (*Ursus thibetanus*)

Analyte^a^	RR^b^	Unit	Group^c^	*n*	Mean	Median	Range (min-max)	SD^d^
ALT	0–109	U/L	BE	243	66.8	52	(7–342)	48.78
			FNE	95	46.7	43	(10–304)	34.53
			ZOO	20	36.1	31.5	(13–99)	20.21
			FR	25	43.4	41.0	(21–98)	18.05
ALKP	0–121	U/L	BE	226	58.2	34.6	(7–1464)	110.29
			FNE	95	44.7	41.0	(10–54)	22.28
			ZOO	20	124.7	118.0	(59–154)	45.99
			FR	25	49.3	47.0	(15–100)	23.28
GGT	72–114	U/L	BE	220	263.4	135	(6–2093)	320.8
			FNE	82	106	63.0	(20–720)	129
			ZOO	20	23.0	17.7	(7–94)	19.20
			FR	25	19.0	16.0	(9–42)	8.12
LDH	45–510	U/L	BE	186	633.8	475	(228–3608)	533.8
			FNE	66	1081	1101	(261–1920)	468
			ZOO	20	701.4	672.5	(384–1064)	193.81
			FR	25	902.3	851.0	(550–1293)	202.90
CK	70–94	U/L	BE	185	351.1	125.0	(32–9232)	979
			FNE	71	178	77.0	(5–2569)	345
			ZOO	20	144.7	131.0	(63–430)	79.83
			FR	25	172.6	153.0	(67–471)	99.41
TBIL	0–8.55	μmol/L	BE	231	4.4	1.8	(0.1–71.0)	7.91
			FNE	47	3.4	2.8	(0.3–15.4)	2.88
			ZOO	20	1.7	1.7	N/A	0.00
			FR	25	2.1	1.7	(1.7–5.1)	0.89
BUN	0.8–4.0	mmol/L	BE	242	5.5	4.3	(1.3–52.4)	5.18
			FNE	94	4.9	4.5	(0.8–18.2)	2.31
			ZOO	20	4.3	4.5	(2.5–6.1)	0.94
			FR	25	4.3	3.6	(1.1–13.6)	2.56
CREA	62–221	μmol/L	BE	232	132.1	124.6	(25.5–594.7)	56.55
			FNE	94	127.2	127.5	(48.0–272.3)	38.32
			ZOO	20	122.4	123.8	(79.6–159.1)	22.08
			FR	25	107.8	106.1	(61.9–247.5)	38.19

^a^ALT = alanine transaminase, ALKP = alkaline phosphatase, GGT = gamma glutamyltransferase, LDH = lactate dehydrogenase, CK = creatine kinase, TBIL = total bilirubin, BUN = blood urea nitrogen; CREA = creatinine

^b^RR = reference range [13–15]

^c^BE = bile-extracted, FNE = farm-not-extracted, ZOO = zoo, FR = free-range

^d^SD = standard deviation

**Table 2:** Comparison of serum proteins and hematology parameters across four groups of Asiatic black bears (*Ursus thibetanus*)

Analyte^a^	RR^b^	Unit	Group^c^	*n*	Mean	Median	Range (min-max)	SD^d^
ALB	29–53	g/L	BE	244	41.3	40.9	(23–58.9)	6.35
			FNE	97	32.2	31.0	(14.0–60.7)	6.68
			ZOO	20	30.1	31.0	(18.0–34.0)	3.65
			FR	25	33.0	32.0	(29.0–39.0)	2.61
GLOB	20–49	g/L	BE	244	34.2	33.6	(15.2–76.0)	8.99
			FNE	96	38.0	39.0	(22.3–51.0)	5.73
			ZOO	20	47.2	47.5	(39.0–60.0)	5.13
			FR	25	39.4	39.0	(33.0–47.0)	4.22
TP	60–92	g/L	BE	244	75.5	74.9	(42.5–106.0)	9.08
			FNE	97	70.0	70.0	(44.0–92.0)	7.58
			ZOO	20	77.3	77.5	(57.0–87.0)	6.41
			FR	25	72.4	71.0	(64.0–85.0)	5.39
PCV	30–54	%	BE	230	43.0	43.4	(13.7–67.6)	8.44
			FNE	69	42.8	44.0	(8.0–58.0)	8.38
			ZOO	20	48.8	48.4	(44.0–54.9)	3.15
			FR	24	45.3	45.5	(34.3–63.8)	6.05
TWBC	3.14–10.43	X10^9/L	BE	237	11.23	9.65	(3.20–112.0)	8.95
			FNE	54	7.75	7.40	(2.30–14.50)	2.86
			ZOO	20	11.37	11.75	(6.80–15.0)	2.26
			FR	24	7.48	7.30	(3.40–14.90)	2.53

^a^ALB = albumin, GLOB = globulin, TP = total protein, PCV = packed cell volume, TWBC = total white blood cell

^b^RR = reference range [13–15]

^c^BE = bile-extracted, FNE = farm-not-extracted, ZOO = zoo, FR = free-range

^d^SD = standard deviation

**Fig. 1:**
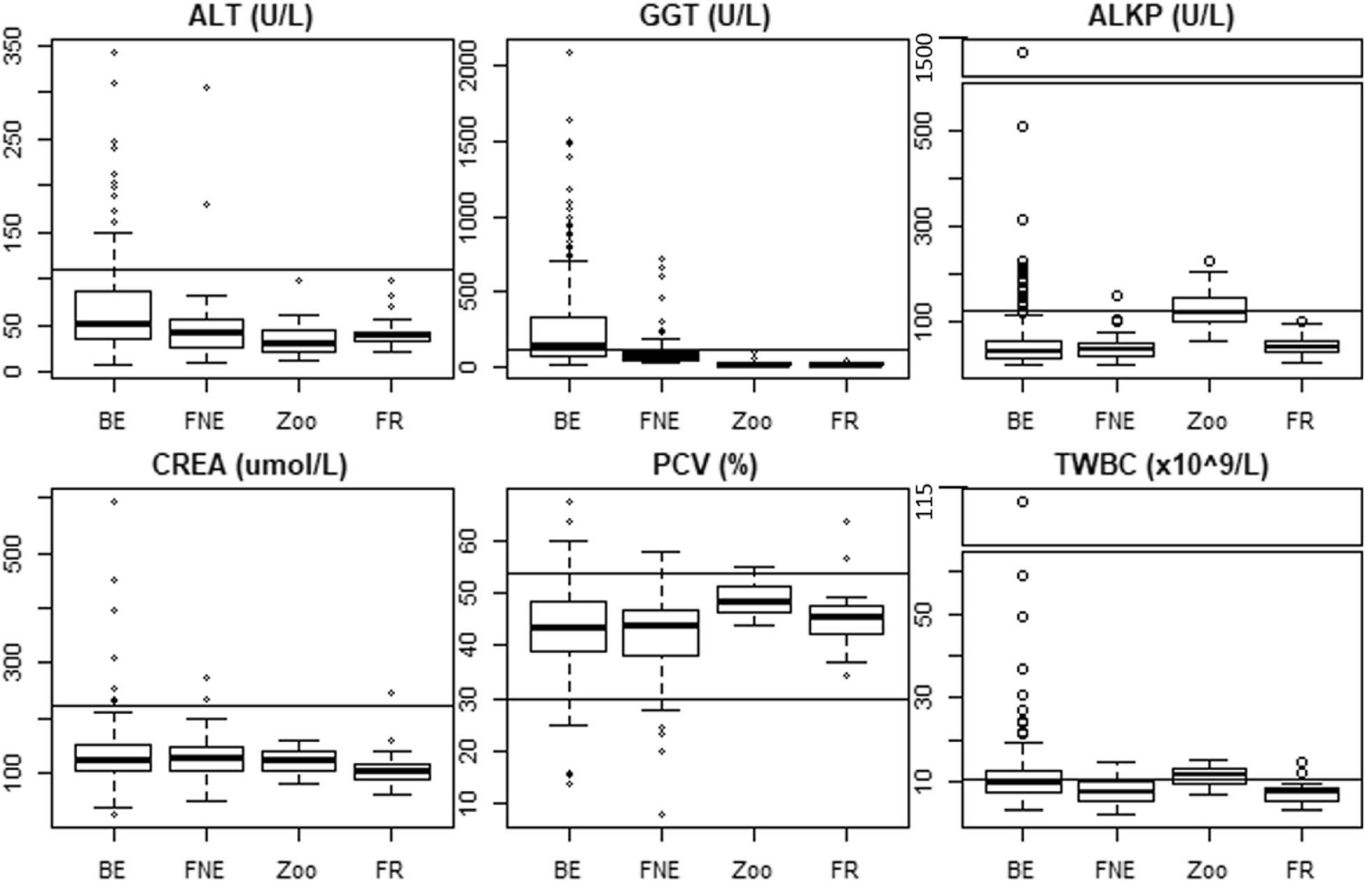
Distribution comparisons of serum parameters across four groups of Asiatic black bears (*Ursus thibetanus*). Sample sizes for each bear group and parameter are listed in Tables 1 and 2. ALT = alanine transaminase, GGT = gamma glutamyltransferase, ALKP = alkaline phosphatase, CREA = creatinine, PCV = packed cell volume, TWBC = total white blood cell, BE = bile-extracted, FNE = farm-not-extracted, FR = free-range. Solid horizontal lines across entire plot for each analyte denote upper reference limit for Asiatic black bears [13–15]. Note split y-axes for ALKP and TWBC. Thick horizontal line within each box represents the median sample value, the bottom and top of each box represents the 25th and 75th quantiles (1st and 3rd quartiles), and whiskers extend to the data point within 1.5 x interquartile range of the 1st and 3rd quartiles, or by the lowest or highest data point

Box and whisker plots displaying distributions of select analytes for each bear group are presented in Fig. 1.

The proportion of BE bears that arrived from bile farms with increased activities of ALT, GGT, ALKP, and TBIL were 15% (36/239), 58% (126/217), 9% (20/222), and 10% (24/230), respectively. Elevated BUN and CREA were present in 57% (136/238) and 3% (7/228), respectively, of BE bears. Leukocytosis (TWBC ≥11 × 10^9^/L) was present in 38.9% (91/234) of BE bears on arrival from bile farms. Only BE bears exhibited TWBC counts greater than 20 × 10^9^/L, although median TWBC was highest in ZOO bears. Differential WBC counts revealed neutrophilia as a predominant reason for elevated TWBC in BE bears. Anemia (PCV < 29%) was present in 5% (12/226) of BE bears. The leading causes of death in bile-extracted bears were hepatic neoplasia (29%, 56/196), paralysis/paresis/deteriorating mobility (28%, 54/196), and cardiovascular disease (11%, 22/196).

Increased activities of ALT, GGT, ALKP, and TBIL were present in 2% (2/99), 17.6% (15/85), 1% (1/99), and 4% (2/48) of FNE bears. Anemia was present in 5% (4/73), and elevations in BUN and CREA in 66% (67/101) and 2% (2/101) of FNE bears on arrival from bile farms.

In comparison, ALT, GGT, TBIL, and PCV were within normal limits for all ZOO bears sampled. Mild increases in ALKP activity and elevations of BUN and TWBC activity were present in 45% (9/20), 65% (13/20), and 55% (11/20) of ZOO bears. Median TWBC was highest for ZOO bears. Differential TWBC counts revealed eosinophilia as a predominant reason for elevated TWBC in ZOO bears.

In the FR bear group, ALT, GGT, TBIL, and ALKP were within normal limits for all bears. Elevated BUN, CREA, and TWBC were present in 48% (12/25), 4% (1/25), and 8.3% (2/24), respectively, of FR bears. Median TWBC was normal for this group and anemia was not present in any FR bears. Two FR bears had leukocytosis with mild neutrophilia.

### Persistent elevations of serum analytes over time

**Fig. 2:**
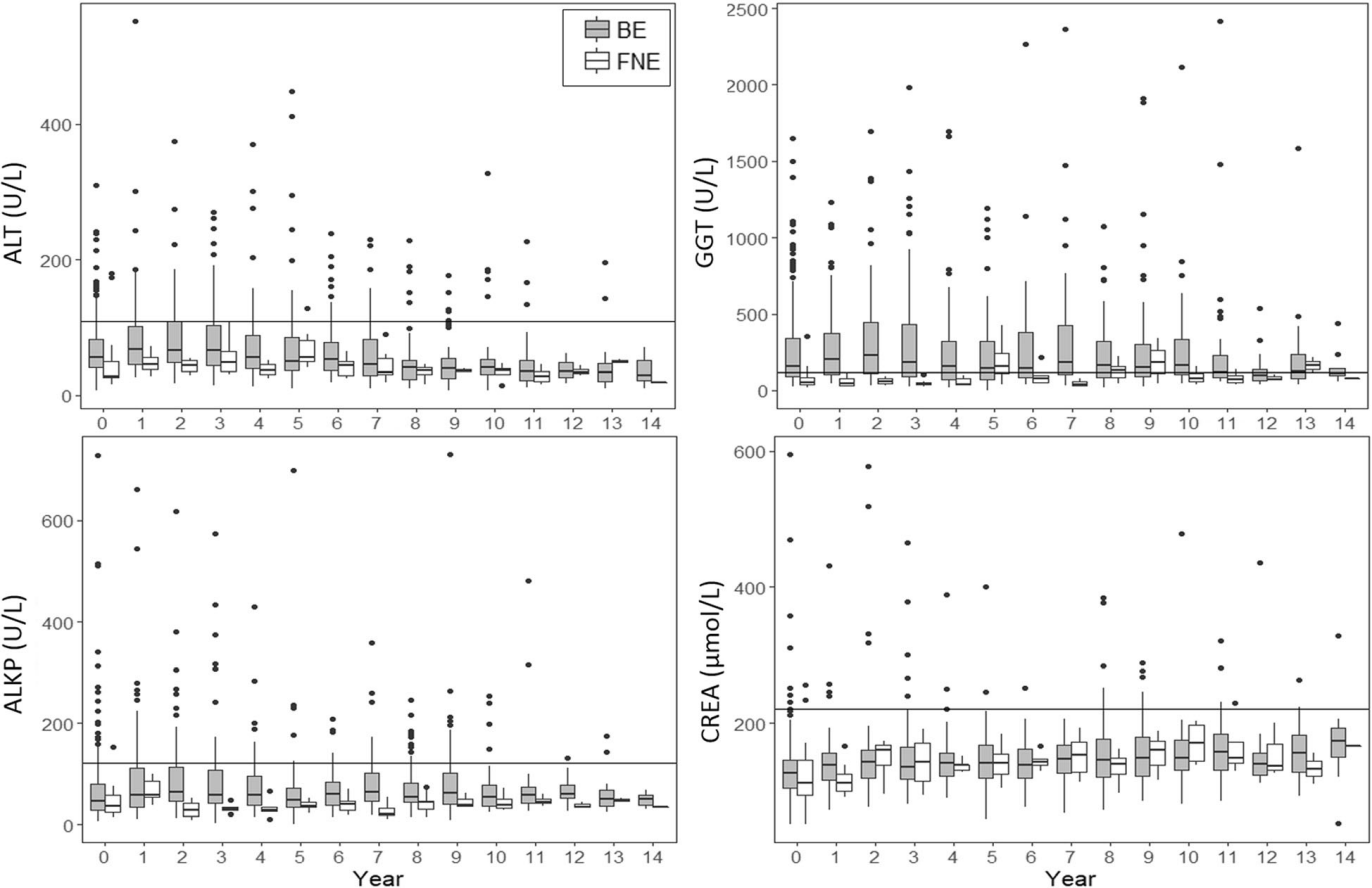
Longitudinal comparison of serum analyte measurements between bile-extracted (*n* = 239) and farm-not-extracted (*n* = 24) bears from day of arrival from bile farms (Year 0) to either death or until 14 years post-rescue demonstrating greater elevations in BE bears that persist over time. ALT = alanine transaminase, GGT = gamma glutamyltransferase, ALKP = alkaline phosphatase, CREA = creatinine, gray boxes = bile-extracted bears, white boxes = farm-not-extracted bears. Note the split y-axis for CREA. Horizontal lines represent the upper limit of normal reference range for Asiatic black bears [13–15]

The BE bear group exhibited long-term elevations (on a population level) and wider ranges of ALT, GGT, ALKP, TBIL, and CREA compared to FNE bears when comparing and tracking serial blood parameter measurements obtained at arrival from bile farms until death or euthanasia, or up to 14 years post-rescue (Fig. 2).

### Effects of age, sex, and/or season on serum biochemical and hematological parameters between bear groups (analysis of covariance (ANCOVA))

When accounting for season, sex, and/or age, significant differences in serum biochemical and hematological parameters were detected between BE and non-farmed, non-extracted bear groups. Estimated differences between categorical levels, standard errors, 95% confidence intervals, and *p*-values from the Analysis of Covariance (ANCOVA) models are presented in Tables 3, 4 and 5, and Additional file 1: Table S1 and S2. A comparison of the effect of age between bear groups for TWBC is shown in Table 4 and Additional file 1: Table S1B.

**Table 3:** ANCOVA Model 2A: Bile-Extracted, Farm-Not-Extracted, Zoo, and Free-Range data adjusting for sex and age (log transformed)

Serum/Hematology Parameters	Obs	Bear Group (*p*-value)	Interactions		Level	Difference	SE	Lower CI	Upper CI	*p-*value
			Sex	Age						
ALT	257	0.0004	–	–	BE-FNE	0.473	0.145	0.182	0.764	0.001^c^
					BE-Zoo	0.552	0.232	0.086	1.018	0.020^c^
					BE-FR	0.357	0.154	0.049	0.665	0.023^c^
GGT	236	< 0.0001	–	–	BE-FNE	1.083	0.204	0.672	1.493	< 0.0001^c^
					BE-Zoo	2.215	0.319	1.575	2.856	< 0.0001^c^
					BE-FR	2.109	0.212	1.683	2.536	< 0.0001^c^
ALKP	239	< 0.0001	–	–	BE-FNE	0.123	0.165	−0.209	0.456	0.480^c^
					BE-Zoo	−1.381	0.260	−1.903	− 0.858	< 0.0001^c^
					BE-FR	−0.139	0.173	−0.486	0.208	0.444^c^
LDH	199	0.0004	–	–	BE-FNE	− 0.078	0.111	− 0.301	0.144	0.5035^c^
					BE-Zoo	−0.345	0.163	− 0.672	− 0.018	0.039^c^
					BE-FR	− 0.433	0.109	−0.653	− 0.214	0.0001^c^
CREA	247		0.0238	–	F,BE-F,FNE	− 0.114	0.089	− 0.386	0.158	0.904^b^
					F,BE-F,Zoo	0.007	0.148	−0.445	0.459	1.000^b^
					F,BE-F,FR	0.281	0.118	−0.080	0.642	0.256^b^
					M,BE-M,FNE	0.217	0.127	−0.171	0.605	0.678^b^
					M,BE-M,Zoo	0.302	0.191	−0.283	0.887	0.763^b^
					M,BE-M,FR	0.035	0.102	− 0.277	0.346	1.000^b^
ALB	259	< 0.0001	–	–	BE-FNE	0.053	0.033	−0.013	0.118	0.115^c^
					BE-Zoo	0.368	0.053	0.262	0.475	< 0.0001^c^
					BE-FR	0.214	0.035	0.143	0.284	< 0.0001^c^
GLOB	258	< 0.0001	–	–	BE-FNE	−0.055	0.054	−0.164	0.053	0.3265^c^
					BE-Zoo	−0.322	0.087	−0.496	− 0.147	0.0003^c^
					BE-FR	−0.218	0.058	−0.334	−0.103	0.0002^c^
PCV	247	0.0299	–	–	BE-FNE	0.068	0.046	−0.025	0.161	0.154^c^
					BE-Zoo	−0.148	0.074	− 0.296	0.001	0.051^c^
					BE-FR	−0.073	0.049	−0.172	0.025	0.146^c^

Obs = number of observations; SE = standard error; CI = confidence interval; ALT = alanine aminotransferase; GGT = gamma glutamyl transferase; ALKP = alkaline phosphatase; LDH = lactate dehydrogenase; CREA = creatinine; ALB = albumin; GLOB = globulin; PCV = packed cell volume; BE = bile-extracted; FNE = farm-not-extracted; FR = free-range; F = female; M = male; Hib = hibernation season (December–April); Non = non-hibernation season (May–November); “- -” indicates *p*-values > 0.05 and denotes interaction terms removed by stepwise selection, resulting in the reported significant p-values listed under Bear Group or Interaction columns after the other interactions terms were removed from the model; ^b^Tukey HSD, ^c^Dunnett

**Table 4:** ANCOVA Model 2A: The difference in the effect of age on log Total White Blood Cell (TWBC). The “Estimate/Difference” is the estimated difference in the effect of age on TWBC between the bear groups specified in the “Level” column (log transformed)

Model	Obs	Bear Group (*p*-value)	Interactions			Level	Estimate/Difference	SE	Lower CI	Upper CI	*p-*value
			Sex	Season	Age						
2A	255		–	–	0.0022	BE-FNE	0.0051	0.0129	−0.0204	0.0305	0.6963
						BE-Zoo	0.0062	0.0137	−0.0208	0.0333	0.6511
						BE-FR	0.0263	0.0189	−0.0110	0.0635	0.1657

“- -” indicates *p*-values > 0.05 and denotes interaction terms removed by stepwise selection, resulting in the reported significant *p*-values listed under Interaction after the other interactions terms were removed from the model

**Table 5:** ANCOVA Model 2B: Bile-Extracted, Farm-Not-Extracted, Zoo, and Free-Range Bear Data adjusting for sex (log transformed)

Serum/Hematology Parameters	Obs	Bear Group (*p*-value)	Interactions	Level	Difference	SE	Lower CI	Upper CI	*p-*value
			Sex						
ALT	383	< 0.0001	–	BE-FNE	0.287	0.074	0.138	0.436	0.000^c^
				BE-Zoo	0.510	0.145	0.220	0.801	0.001^c^
				BE-FR	0.249	0.132	0.015	0.513	0.065^c^
GGT	344	< 0.0001	–	BE-FNE	0.732	0.118	0.493	0.970	< 0.0001^c^
				BE-Zoo	2.093	0.211	1.663	2.523	< 0.0001^c^
				BE-FR	2.128	0.192	1.737	2.519	< 0.0001^c^
ALKP	366	< 0.0001	–	BE-FNE	−0.037	0.084	−0.211	0.137	0.737^c^
				BE-Zoo	−1.135	0.163	−1.471	−0.800	< 0.0001^c^
				BE-FR	−0.111	0.148	−0.417	0.194	0.515^c^
LDH	294	< 0.0001	–	BE-FNE	−0.568	0.070	−0.714	−0.423	< 0.0001^c^
				BE-Zoo	−0.218	0.117	−0.460	0.023	< 0.0001^c^
				BE-FR	−0.471	0.107	−0.691	− 0.250	0.078^c^
CK	301	0.0002	–	BE-FNE	0.593	0.131	0.318	0.867	< 0.0001^c^
				BE-Zoo	0.209	0.222	−0.256	0.675	0.424
				BE-FR	0.091	0.202	−0.334	0.516	0.761
CREA	371		< 0.0001	F,BE-F,FNE	−0.134	0.052	−0.294	0.025	0.172^b^
				F,BE-F,Zoo	−0.077	0.103	−0.390	0.237	0.996^b^
				F,BE-F,FR	0.309	0.113	−0.036	0.653	0.117^b^
				M,BE-M,FNE	0.225	0.061	0.039	0.412	0.006^b^
				M,BE-M,Zoo	0.164	0.115	−0.186	0.515	0.843^b^
				M,BE-M,FR	0.150	0.089	−0.122	0.422	0.701^b^
TP	386	< 0.0001	–	BE-FNE	0.074	0.014	0.046	0.101	< 0.0001^c^
				BE-Zoo	−0.027	0.027	−0.081	0.026	0.328^c^
				BE-FR	0.033	0.024	−0.016	0.082	0.186^c^
ALB	386	< 0.0001	–	BE-FNE	0.253	0.020	0.214	0.293	< 0.0001^c^
				BE-Zoo	0.311	0.038	0.234	0.388	< 0.0001^c^
				BE-FR	0.207	0.035	0.137	0.277	< 0.0001^c^
GLOB	385	< 0.0001	–	BE-FNE	−0.127	0.027	−0.181	−0.073	< 0.0001^c^
				BE-Zoo	−0.350	0.053	−0.456	−0.245	< 0.0001^c^
				BE-FR	−0.172	0.048	−0.268	−0.076	0.0004
PCV	343	0.0148	–	BE-FNE	0.009	0.030	−0.052	0.070	0.790^c^
				BE-Zoo	−0.150	0.053	−0.256	− 0.044	0.005^c^
				BE-FR	−0.077	0.049	−0.175	0.022	0.128^c^
TWBC	335	< 0.0001	–	BE-FNE	0.314	0.062	0.189	0.439	< 0.0001^c^
				BE-Zoo	−0.113	0.098	−0.310	0.083	0.2651^c^
				BE-FR	0.354	0.091	0.171	0.536	0.0001^c^

Obs = number of observations; SE = standard error; CI = confidence interval; ALT = alanine aminotransferase; GGT = gamma glutamyl transferase; ALKP = alkaline phosphatase; LDH = lactate dehydrogenase; CK = creatine kinase; CREA = creatinine; TP = total protein; ALB = albumin; GLOB = globulin; PCV = packed cell volume; TWB = total white blood cell count; BE = bile-extracted; FNE = farm-not-extracted; FR = free-range; F = female; M = male; “- -” indicates *p*-values > 0.05 and denotes interaction terms removed by stepwise selection, resulting in the reported significant p-values listed under Bear Group or Interaction columns after the other interactions terms were removed from the model; ^b^Tukey HSD, ^c^Dunnett

Model 1 (BE and FNE bear data) was run to assess the effect of season on serum biochemical and hematology parameters. Age estimates were not available for all bears, so to assess whether model output/conclusions were sensitive to missing age data, two sub-models were run: Model 1A adjusted for sex, season, and age (BE and FNE bears (Additional file 1: Table S1A,B) and Model 1B excluded age and adjusted for sex and season only (BE and FNE bears (Additional file 1: Table S2). Only two biochemical parameters, albumin (ALB) and lactate dehydrogenase (LDH) had a significant season interaction, where larger differences in mean ALB and LDH were found between BE and FNE bears in winter with no evidence for differences in summer. ALB and LDH were not considered critical parameters for this study, therefore we opted to focus on Model 2.

Model 2 allowed for comparisons across all four bear groups adjusting for sex and age (Model 2A, Tables 3 and 4) or sex only (Model 2B, Table 5) to assess whether model output and conclusions were sensitive to missing age data (excluding season as ZOO and FR bears were only sampled in summer months). The only parameter with a significant age interaction was TWBC (Model 2A, Table 4) with older ages indicating higher TWBC in BE bears than FNE bears. There was a significant sex interaction for CREA, with greater differences between male BE and male ZOO and FNE bears, and female BE and female FR bears, but post-hoc analyses were not significant.

Age was not known for a substantial number of bears and by excluding this covariate from the ANCOVA model, more data points were included which resulted in more degrees of freedom, and also resulted in an increase in the number of analytes where bear groups were detected as distinct from one another. This suggests that bear group differences in some analytes were sensitive to the presence/absence of missing age data. There were some discrepancies in terms of significant interactions between covariates. These differences could be due to bias in the group with missing age data, the effect of age, or the change in sample size. The minor discrepancies did not affect the overall conclusions for those parameters most relevant to bile-extraction, therefore Model 2B was chosen to focus on all available data.

For Model 2B (BE, FNE, ZOO, and FR bears adjusting for sex (Table 5)), BE bears had greater mean GGT and ALB compared to all other bear groups, greater mean ALT than FNE and ZOO bears, and greater mean creatine kinase (CK) and total protein (TP) than FNE bears. Mean TWBC was higher in BE and ZOO compared to FNE and FR bears. There was a significant sex interaction for CREA, with higher CREA in male BE vs male FNE bears. BE bears had lower mean globulin (GLOB) than all other bear groups and lower mean LDH than FNE and ZOO bears. FNE bears had greater mean GGT compared to ZOO and FR bears and greater mean LDH compared to FR bears. FNE bears had lower mean PCV compared to ZOO bears. In contrast, ZOO bears had higher mean ALKP compared to BE and FNE bears.

### Total bilirubin in BE compared to non-extracted bears (logistic regression)

The odds of having elevated TBIL was 7.3 times greater in BE bears compared to all non-extracted bear groups combined (95% Confidence Interval (CI) 1.7 to 31.3).

### Reduced survival in BE compared to FNE bears

**Fig. 3:**
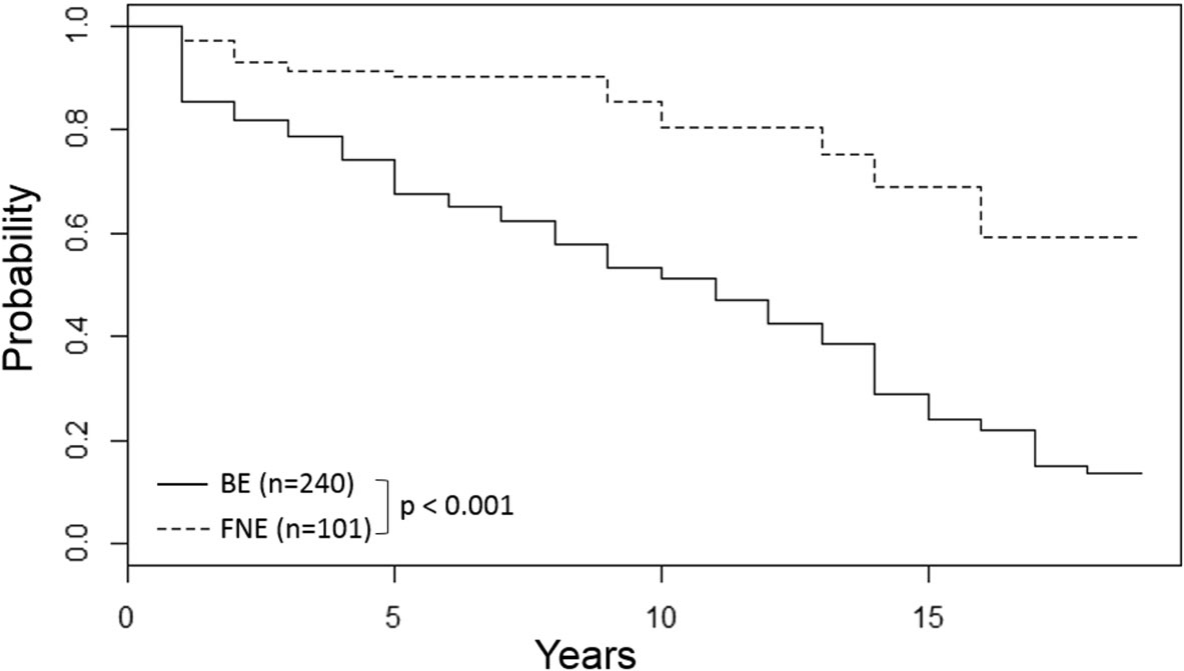
Kaplan Meier survival analysis for bile-extracted (BE) (*n* = 240) compared to farm-not-extracted (FNE) (*n* = 101) Asiatic black bears (*Ursus thibetanus*). *p* < 0.05, logrank test

Kaplan Meier survival analysis showed significantly shorter survival times for the BE group with a median survival time of 11 years post-rescue compared to over 17 years for the FNE group (*p* < 0.05, logrank test) (Fig. 3). Cox proportional hazards regression showed age contributed significantly to survival; with each additional year of age, the daily hazard of death increased by a factor of 1.12 or 12% (Hazard Ratio (HR) 1.12, 95% CI (1.07, 1.17), *p* < 0.05).

**Fig. 4:**
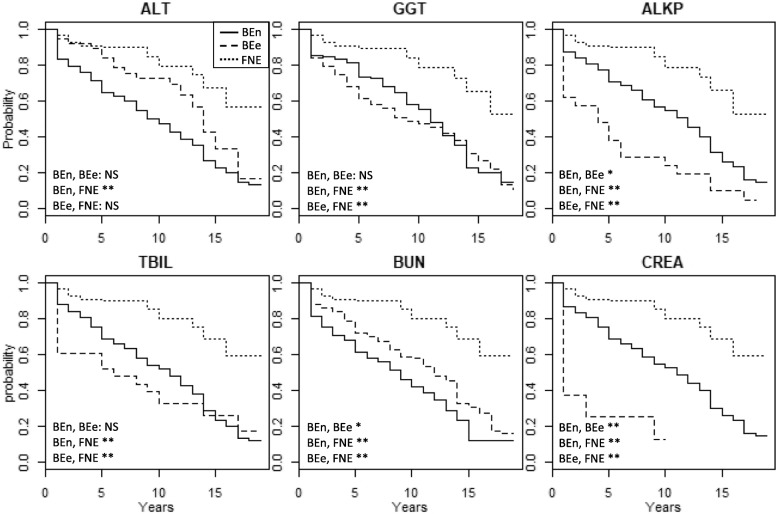
Kaplan Meier survival analysis of bile-extracted bears with normal or elevated analyte values and farm-not-extracted bears. ALT = alanine transaminase, GGT = gamma glutamyltransferase, ALKP = alkaline phosphatase, TBIL = total bilirubin, BUN = blood urea nitrogen, CREA = creatinine, BEn = BE bears with normal activities/values; BEe = BE bears with elevated activities/values; NS = not significant; * indicates *p*-value < 0.05, ** indicates *p*-value < 0.001. Log rank test was used to determine statistical significance between curves. Sample sizes: ALT BEn (*n* = 202), BEe (*n* = 37), FNE (*n* = 100); GGT BEn (*n* = 91), BEe (*n* = 126), FNE (*n* = 85); ALKP BEn (*n* = 200), BEe (*n* = 21), FNE (*n* = 99); TBIL BEn (*n* = 207), BEe (*n* = 23), FNE (n = 101); BUN BEn (*n* = 103), BEe (*n* = 135), FNE (*n* = 101); CREA BEn (*n* = 226), BEe (*n* = 8), FNE (*n* = 101)

BE bears that arrived from bile farms with increased serum activities/levels of ALKP and CREA had markedly shorter median survival times of 4 and 1 year(s) post-rescue, respectively (Fig. 4) compared to 11 years for BE bears that arrived with normal serum activities/levels of the same analytes. Regardless of serum activities/levels of ALKP, GGT, TBIL, BUN, or CREA, all BE bears had shorter survival times compared to FNE bears (*p* < 0.01, logrank test) (Fig. 4).

## Discussion

Four groups of Asiatic black bears were used for this study, one bile-extracted group (BE) and three non-extracted groups (FNE, ZOO, and FR). Bears that were bile extracted had more abnormalities in serum biochemical and hematological parameters than those that were not bile-extracted. Many of these derangements were persistent over time and were associated with reduced long term survival when compared to non-extracted bears.

Higher serum biochemical values (i.e. ALT and GGT) of BE bears on arrival from bile farms are consistent with hepatobiliary disease [16–18]. When followed over time, increases in serum ALT, GGT, and ALKP activity in the BE group persisted throughout the period of analysis and are consistent with long-term hepatobiliary effects in bears who previously experienced bile extraction (Fig. 2). In contrast, ZOO and FR bears did not have evidence of hepatobiliary disease as both groups had normal ALT and GGT values.

Hepatobiliary disease would be expected in BE bears as they undergo gallbladder fistulation and/or catheterization which induces tissue trauma and provides a portal for foreign material and bacteria to enter the gallbladder, ascend the biliary tree, and affect hepatocytes. This can result in chronic cholecystitis/cholangitis/cholestasis, and subsequent inflammatory/infectious dissemination into the abdomen and systemic circulation. Not surprisingly, pathologies associated with bile extraction sites of rescued bears include abdominal hernias, peritoneal abscesses/peritonitis, cholelithiasis, gallbladder polyps [6], and a high prevalence of cholecystitis (73.1–99.5%) (Wendi Roe pers. comm.). Positive bacterial cultures were detected in 85% (60/71) of samples of bile and gallbladder tissues of BE bears (animal medical records review). Increases in serum ALT activities can also be seen with extrahepatic conditions such as muscle injury, peritonitis, septicemia, and chronic inflammation and these conditions are commonly observed in BE bears [6, 16, 17, 19]. Drug-induced increases in liver enzyme activities cannot be ruled out as medications, particularly antibiotics, are known to be administered to bears on bile farms, although records from bile farms are often incomplete or unavailable [6, 11]. Anemia as well as elevated serum activities of ALT and GGT were present in a small proportion of FNE bears but not in other non-extracted bear groups. This finding suggests that FNE bears on bile farms are subjected to the same poor husbandry and extrahepatic conditions as noted previously.

Interestingly, mean ALKP was similar between BE, FNE, and FR bears and the proportion of BE bears with elevated ALKP was small. Increased serum activity of ALKP is typically seen with hepatobiliary disease in other species and would be expected to be observed in BE bears. BE bears exhibited the highest individual serum ALKP activity among all bear groups (Table 1, Fig. 1) and these BE bears also had very high GGT activities. These results suggest that GGT is a more sensitive indicator of hepatobiliary disease than ALKP in Asiatic black bears undergoing bile extraction as the proportion of BE bears with elevations in serum GGT activity was greater than the proportion of BE bears with elevations in serum ALKP activity.

ZOO bears demonstrated the highest mean ALKP and the highest proportion of bears with increased ALKP activity of the four bear groups. Systemic disease, drugs, endogenous corticosteroids, and increased osteoblastic activity in young growing animals can also increase serum ALKP activity [16, 17, 19]. Age data were available for 4/9 ZOO bears and all of these were young (1 year old) animals at the time of sampling, and all other liver enzymes were within normal limits. In similar bear species, depending on sex, 90% of skeletal maturity is achieved at 3.5 years of age and 97% at 5 years of age [20]. Therefore, increased ALKP activity in young ZOO bears in this study is consistent with increases in the bone isoenzyme of ALKP in this group of bears.

BE bears exhibited the widest range (on a population level) and some of the highest individual values of CREA on arrival from bile farms and elevations persisted over time (Figs. 1 and 2), consistent with long term renal effects in BE bears that were not evident in FNE bears. Elevated serum CREA can be an indicator of renal disease, although elevations may be pre-renal (dehydration/hemoconcentration) in origin [21]. Urinary obstruction (post renal) was not reported in any of the bear groups. FR bears had limited access to water when in barrel traps, and thus a degree of hemoconcentration could explain similar CREA values between BE and FR bears.

Lack of free access to water has been previously reported for bears on bile farms, which would promote volume contraction [6, 8]. Because husbandry conditions and water access would likely be similar for both BE and FNE bears housed on bile farms, the elevated CREA in BE bears is suspected to indicate renal disease. The primary difference between BE and FNE bears is presence of bile extraction sites that are sources of chronic infection that could spread hematogenously resulting in subsequent renal disease such as pyelonephritis, or possibly immune complex deposition as a result of chronic inflammation. Unfortunately, urine samples were not obtained in newly rescued bears from bile farms, therefore it was not possible to compare serum BUN and CREA with urine specific gravity to determine whether azotemia was pre-renal or renal in nature. Concurrent volume contraction and renal disease could also be possible in this population of bears. However, persistent elevation in CREA over time in BE bears (and not in FNE bears) suggests the presence of chronic renal disease in the BE group.

Serum CREA is also affected by muscle mass, and low serum CREA can be seen with reduced muscle mass/muscle wasting [21, 22]. 90% of bears rescued from bile farms were reported to be underweight or emaciated on arrival by staff veterinarians, and thus it is plausible that hydration status and renal function are not accurately assessed by serum CREA values in BE bears. ZOO and FR bears in this study were in good body condition. Poor muscle mass and smaller body size could also contribute to FNE bears and female BE bears having lower CREA compared to ZOO and FR bears.

On arrival from bile farms, BE bears had significantly higher mean ALB and lower mean GLOB than all other bear groups. Hyperalbuminemia is most commonly due to dehydration, which, as discussed earlier, is possible due to restricted access to water on bile farms. Hyperalbuminemia has also been associated with hepatocellular carcinoma [23]. Hypoglobulinemia is commonly due to loss of globulins (hemorrhage or enteric loss), or lack of globulin production (liver disease and cachectic states) [24]. Globulin is synthesized in the liver and thus hypoglobulinemia is congruent with the high prevalence of liver disease in the BE group.

Given that albumin is affected by protein intake and is a negative acute phase protein, we might expect to see a higher prevalence of hypoalbuminemia in BE bears due to suspected protein deficient diets and presence of chronic inflammation/infection [25, 26]. However concurrent dehydration/hemoconcentration may mask hypoalbuminemia (and overestimate GLOB) in BE bears, similar to findings in starved dogs [25]. Offering explanations for changes in serum proteins is challenging due to competing influences to raise or lower serum concentrations of both albumin and globulin in BE bears.

Both BE and FNE bears exhibited the widest range in PCV, in the form of anemia as well as erythrocytosis. Conditions resulting in increased loss and/or decreased production of erythrocytes are blood loss, nutritional deficiencies, liver failure, renal disease, and anemia of chronic inflammatory disease, all of which have been described in BE bears. Of note, 14 BE bears exhibited anemia on arrival from bile farms and six of these bears were euthanized within nine days of rescue due to hepatic neoplasia, and two bears died of peritonitis within five months of arrival. Four FNE bears arrived with anemia and one of these bears died within three months of rescue with findings of emaciation, enteritis, and parasitism. Two of the FNE bears with anemia were young bears (< 1 year), consistent with iron deficiency anemia secondary to growth demands in young animals that has been previously documented in bears [27, 28]. Concurrent hemoconcentration may also mask the presence of anemia, and anemia in adult bears on arrival from bile farms may be considered a poor prognostic sign.

The greater range and magnitude of leukocytosis in BE bears was explained by neutrophilia and is consistent with infection and inflammation of bile extraction sites when arriving from bile farms. BE bears may also have a component of stress neutrophilia in response to conditions on bile farms, transport from bile farms to sanctuary, and/or secondary to illness as seen in small animals [29]. Leukocytosis secondary to stress is plausible in this group of bears as previous studies have documented elevated cortisol levels in bears on farms and in bears immediately upon arrival at the rescue center when compared to rehabilitated bears sampled over 163 days post-rescue [30]. ZOO bears exhibited leukocytosis characterized by eosinophilia, presumably due to parasitism, although allergic responses cannot be ruled out. ZOO bears in this study were housed in densely populated groups posing a high risk of parasite exposure and transmission. In contrast, bears on bile farms are confined in solitary cages raised off the ground to facilitate bile extraction, and therefore have fewer opportunities for parasite transmission.

### Survival analysis of bile-extracted bears

Shorter survival times in BE bears compared to FNE bears is consistent with clinical findings of multiple, concurrent health problems observed when BE bears are rescued from bile farms. Age was shown to have a significant impact on survival, with increasing age there was an increase in risk of death. Mean age of BE bears was higher than FNE bears (9 vs 5 years, respectively) and while age would be expected to affect survival probabilities, the older age of BE bears is also associated with longer duration of exposure to bile-extraction practices and associated risks that would be expected to impact health and survival. While FNE bears may also have evidence of compromised health, FNE bears do not experience similar exposure to risks associated with bile extraction, nor do they develop a high incidence of chronic liver or kidney disease, or hepatic neoplasia which could explain their longer survival times.

Increased levels of serum ALKP and CREA on arrival from bile farms were particularly strong predictors of survival in BE bears despite their overall low prevalence in this group. For example, 9% of BE bears arrived with elevated ALKP and had a significantly shorter median survival of 4 years, compared to 11 years in BE bears who arrived with normal ALKP (Fig. 4). Elevations of ALKP could be a marker of hepatic neoplasia as 50% of bears that arrived with elevated ALKP died of hepatic neoplasia, the leading cause of death in BE bears [6, 10].

BE bears that arrived from bile farms with elevated serum CREA levels had a median survival time of 1 year compared to 11 years and over 17 years for bears with normal CREA levels and FNE bears, respectively (Fig. 4). Similar to ALKP, even though the percentage of BE bears with azotemia on arrival was small, azotemia was a strong predictor of early death. This finding combined with elevated CREA in BE bears over time suggests that BE bears may have an increased incidence of renal disease beyond what routine biochemical panels can detect.

Arriving from bile farms with normal serum ALT, GGT, ALKP, TBIL, BUN, and CREA was not protective as BE bears still had significantly shorter survival times than FNE bears (median 11 years vs over 17 years). This is likely due to the presence of milder forms of underlying systemic derangements that were not detected by routine laboratory parameters. These findings underscore the insensitive nature of routine laboratory parameters to identify underlying disease in Asiatic black bears undergoing bile extraction.

## Conclusions

Our results provide strong evidence that bile extraction practices not only represent a temporary constraint for bears’ welfare, but confer distinct long-term adverse health consequences. Regardless of laboratory parameters, BE bears have significantly reduced survival compared to FNE bears. Routine laboratory panels may be insensitive to detect underlying illness in bile-extracted bears as these bears arrive with multiple concurrent problems, such as dehydration and emaciation that may mask the detection of systemic disease. Additional biomarkers, such as symmetric dimethylarginine (SDMA) as a marker for renal function, are needed to assess disease in this population of bears.

## Methods

Due to limited laboratory references for Asiatic black bears, serum biochemical and hematological data from non-farmed, non-extracted Asiatic black bears were obtained to provide a baseline for comparison with data from bile-extracted bears. Zoo and free-range Asiatic black bears in Japan were the closest subspecies available for comparison.

### Study populations

The study included four populations of Asiatic black bears: 244 bile-extracted (BE) (151 females and 93 males), 97 farm-not-extracted (FNE) (54 females and 43 males), 20 zoo (ZOO) (11 females and 9 males), and 25 free-range (FR) (9 females and 16 males) bears (Table 6). Serum biochemical and hematological values were retrospectively analyzed from BE and FNE bears rescued between 2000 and 2014. BE bears were defined as bears with overt evidence of bile extraction including the presence of indwelling abdominal catheters, free-drip fistulas, and/or midline abdominal fibrosis consistent with previous bile-extraction. FNE bears were defined as bears rescued from bile farms with no evidence of bile extraction and suspected to have been used for breeding purposes or not yet undergone surgeries to secure access to the gallbladder. Serial blood samples were obtained from BE and FNE bears starting from the time of their rescue until their death or euthanasia, or until 2014. BE and FNE bears were sampled during routine, biennial health checks, and each time they were anesthetized for any health concerns, which, for some bears, resulted in multiple blood samples obtained within a single year. ZOO bears were located at Ani Mataginosato Bear Park in Akita Prefecture, Japan, and serum biochemical and hematological values for this study were obtained during health examinations conducted in November 2014. FR bears were captured and re-released in the Ashio-Nikko mountains in Tochigi and Gunma prefectures, lakeside areas of Nikko, and the Okutama mountains in Tokyo prefecture, Japan, as part of ongoing field studies [31–33]. Samples were opportunistically taken during these captures between May and November in 2014 and 2015. ZOO and FR bears were sampled only once.

**Table 6:** Descriptive statistics of the study population. Frequency (%) and mean age (range and number of bears with age data) at arrival from bile farms and initial sampling (BE and FNE bears) and at time of sampling (ZOO and FR bears) are reported

		Females (%)	Males (%)	Mean age (yrs) (range)
Bile-extracted	(*n* = 244)	151 (62)	93 (38)	9 (3–30, *n* = 218)
Farm-not-extracted	(*n* = 97)	54 (56)	43 (44)	5 (1–18, *n* = 25)
Zoo	(n = 20)	11 (55)	9 (45)	7 (1–20, *n* = 11)
Free-range	(n = 25)	9 (36)	16 (64)	7 (3–15, *n* = 18)

Most commonly, BE, FNE, and ZOO bears were induced using a combination of zolazepam-tiletamine (Zoletil 100, Virbac, Bury St Edmonds, Suffolk IP30 9UP, England) and medetomidine (Domitor, 1 mg/ml, Zoetis, London EC4A 3AE, England) at dosages ranging from 1 to 3 mg/kg (Zoletil) combined with 0.04 to 0.1 mg/kg (Domitor) administered intramuscularly via pole syringe or blowpipe. BE and FNE bears were intubated and maintained on 1–3% isoflurane as described previously [9]. FR bears were captured and re-released using barrel traps that were baited with honey, anesthetized with zolazepam-tiletamine (Zoletil, Virbac, Carros, France) at an average dose of 7.3 mg/kg, administered via blowpipe as described previously (one FR bear was anesthetized using a combination of zolazepam-tiletamine (2.6 mg/kg) and medetomidine (0.03 mg/kg) as described above) [33]. Age estimates were determined by cementum annuli analysis of pre-molar teeth that were removed under general anesthesia as part of other research protocols for determining age [34, 35]. Handling of BE and FNE bears was part of routine veterinary care performed by registered veterinary staff. Handling of bears in Japan followed the guidelines of the American Society of Mammologists and the Mammalogical Society of Japan [33].

### Sampling procedure

Blood was collected from a jugular vein and placed in 2 mL ethylenediaminetetraacetic acid (EDTA) tubes for hematology and 5 mL serum separator tubes for serum biochemistry analysis. For BE and FNE bears, blood samples collected between 2000 and 2009 were sent to a local human hospital for serum biochemical and hematological analysis. Samples collected after 2009 were analyzed on-site using the Idexx VetTest Chemistry Analyzer (Idexx Laboratories, Inc. Westbrook, Maine 04092, U.S.) within an hour of blood collection, and total white blood cell counts (TWBC) and differentials were performed manually using standard techniques [36].

Blood samples from ZOO and FR bears were kept refrigerated or chilled, respectively, and transported chilled within the same day of collection to a laboratory in Tokyo (Monoris Co., Ltd., Tokyo 182–0012, Japan) for serum biochemical analysis (BioMajesty Series 1650, Jeol, Ltd., Tokyo 196–8558, Japan) and complete blood count (CBC) (Celltac MEK-6358, Nihon Kohden, Tokyo 161–8560, Japan).

Standard serum biochemical panels included alanine transaminase (ALT), gamma-glutamyl transferase (GGT), alkaline phosphatase (ALKP), total bilirubin (TBIL), lactate dehydrogenase (LDH), creatine kinase (CK), blood urea nitrogen (BUN), creatinine (CREA), total protein (TP), albumin (ALB), and globulin (GLOB). A complete blood count (CBC) was also performed. Reference ranges for reported analytes were based on the 2013 International Species Information System (ISIS) Physiological Reference Intervals for Asiatic black bears, and for GGT, LDH, and CK, which were not reported in ISIS, reference intervals were based on Yang et al. [13–15].

### Statistical analysis

Microsoft Excel (2013) was used to compute descriptive statistics (means, medians, ranges, standard deviations). R, Version 3.5.0 (R Core Team 2018) [37], RStudio, Version 1.1.453 (RStudio Team 2016) [38] was used to create box and whisker plots (*plotrix* package) [39], longitudinal plots (*ggplot2* package) [40], and to perform Kaplan Meier survival analysis (*survival* package) [41]. JMP, Version Pro 12 (SAS Institute Inc., Cary, NC, 1989–2007) was used to perform analysis of covariance (ANCOVA) and post-hoc comparisons.

### Descriptive statistics

Descriptive statistics (means, medians, ranges, standard deviations) were used to compare serum biochemical analytes and hematological parameters across bear groups. For this retrospective, cross-sectional comparison, all data obtained from ZOO and FR bears were included, and for BE and FNE bears only blood samples obtained at the time of arrival/rescue from bile farms (between 2000 and 2013) were included as these values were presumed to best reflect the health status of these bears on bile farms. Not all biochemical analytes were measured in every blood sample obtained, therefore different analytes have different sample sizes.

Data from serial blood sample analysis were available from BE and FNE bears from the time of rescue from bile farms to the time of death or euthanasia, or up to 14 years post-rescue. A retrospective, longitudinal analysis utilizing serial measurements from BE and FNE bears was performed to determine whether initial abnormalities in serum biochemical and hematological parameters normalized post-rehabilitation and to compare changes in analyte values over time between BE and FNE bears. When multiple blood samples were obtained from an individual bear within a single year, analyte values were averaged to avoid skewing data towards the lowest or highest value. For most bears, the range of values obtained within a single year were fairly consistent.

### ANCOVA

Analysis of covariance was used to determine whether significant differences existed in serum biochemical and hematological parameters between bile-extracted and non-extracted bears when adjusting for covariates such as sex, age, and/or season. For BE and FNE bears, only values obtained on arrival from bile farms were used for analysis while all available data from ZOO and FR bears were used. The distributions of analytes, assessed using histograms, quantile plots, and the Shapiro Wilk test, were skewed, hence, we applied natural log transformations prior to modeling and no major deviations from normality were detected. Two main ANCOVA models were fit, one accounting for season, and the other excluding season as seasonal comparisons were only available for BE and FNE bears. This model explored whether there were any seasonal effects on blood parameters irrespective of actual hibernation status as BE and FNE bears do not hibernate on bear bile farms. In addition, age data were not available for all bears in all bear groups, therefore, each model was applied twice: once to include age and again to exclude age to assess whether the results of the model were sensitive to the missing age data.

**Fig. 5:**
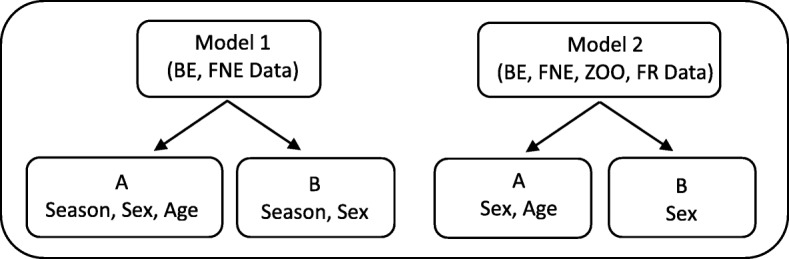
Schematic of the two ANCOVA models applied to different data sets adjusting for different covariates. BE = bile-extracted, FNE = farm not-extracted, ZOO = zoo, FR = free-range

ANCOVA Model 1 used data from BE and FNE bears only and adjusted for season and sex, with age either included or excluded (Fig. 5, Models 1A and B). For simplicity, ‘Season’ for this study was categorized as either “winter” to include samples obtained between December and April when bears are typically inactive, or “summer” to include samples obtained between May and November when bears are typically active. Effects of season were only explored in BE and FNE bears as they were rescued and subsequently sampled throughout the year, whereas ZOO and FR bears were only sampled during the summer season. ANCOVA Model 2 used data from BE, FNE, ZOO and FR bears and adjusted for sex with age either included or excluded (Fig. 5, Models 2A and B).

We applied reverse stepwise selection on the interaction terms, for example, ‘season*bear groups’, ‘sex*bear groups’, and ‘age*bear groups’ (for Model 1A), removing the least significant term (smallest F-value) until any remaining interactions were significant (*p*-value < 0.05), or all interaction terms were removed and only first order terms (bear group, season, sex, and age) remained. Post-hoc comparisons were performed using a student *t*-test (when there was significance at bear group level for Model 1, comparing BE and FNE only), a Tukey Honest Significant Difference (HSD) test (when there was significance at an interaction level for either Model), or a Dunnet’s test (when there was significance at bear group level for Model 2 (comparing BE to all other bear groups)). A *p*-value of < 0.05 was considered significant.

### Logistic regression

A logistic regression analysis was performed to assess for differences in total bilirubin (TBIL) between bear groups. The majority of ZOO and FR bears had total bilirubin (TBIL) values of 1.71 μmol/L, which was the lower limit of detection. Therefore, TBIL values for all bears were converted to dichotomous values, “0” representing values below, and “1” representing values above the upper limit of the reference range (8.55 μmol/L) [13]. FNE, ZOO, and FR bear groups were combined into a “non-extracted” bear group and compared to BE bears to compute an odds ratio.

### Survival analysis

To compare overall survival of BE and FNE bears, Kaplan-Meier survival analysis was performed. Cox proportional hazards regression was used to analyze the effect of age on survival. To determine whether increased serum activities/values of selected biochemical analytes or hematological parameters were associated with reduced survival, Kaplan Meier survival analysis was performed comparing BE bears that arrived with normal vs increased serum activities/values (above the reference range), and FNE bears. Survival data were available from 2000 to 2018. The logrank non-parametric test was used to assess differences in survival over time between BE and FNE bear groups.

## Additional file

Additional file 1: **Table S1A**. ANCOVA Model 1A: Bile-Extracted and Farm-Not-Extracted data adjusting for sex, season, and age (log transformed). The Difference is the estimated average difference in serum parameter (dependent variable) between the bear groups specified in the “Level” column. **Table S1B**. ANCOVA Model 1A: The difference in the effect of age on log Total White Blood Cell (TWBC). The “Estimate/Difference” is the estimated difference in the effect of age on TWBC between the bear groups specified in the “Level” column (log transformed). **Table S2**. ANCOVA Model 1B: Bile-Extracted and Farm-Not-Extracted data adjusting for sex and season (log transformed). Additional file tables provided for additional reference to results from the Analysis of Covariance (ANCOVA) models performed to assess for differences in serum biochemical and hematological parameters when accounting for season, sex, and/or age between BE and non-farmed, non-extracted bear groups. Estimated differences between categorical levels, standard errors, 95% confidence intervals, and *p*-values from the Analysis of Covariance (ANCOVA) models are presented in Tables 3, 4 and 5, and Table S1 and S2. A comparison of the effect of age between bear groups for TWBC is shown in Table 4 and Table S1B. (DOCX 24 kb)

## Data Availability

The datasets used and/or analyzed during the current study are available from the corresponding author on reasonable request.

## Abbreviations

ALB: Albumin
ALKP: Alkaline phosphatase
ALT: Alanine transaminase
ANCOVA: Analysis of covariance
BE: Bile-extracted
BUN: Blood urea nitrogen
CBC: Complete blood count
CI: Confidence interval
CK: Creatine kinase
CREA: Creatinine
FNE: Farm not-extracted
FR: Free range
GGT: Gamma-glutamyl transferase
GLOB: Globulin
LDH: Lactate dehydrogenase
TBIL: Total bilirubin
TP: Total protein
Tukey: HSD Tukey Honest Significant Difference test
TWBC: Total white blood cell count
Yr: Year
