# Supplementary material for: Metabolic derangements and reduced survival of bile-extracted Asiatic black bears (Ursus thibetanus)
Source: BMC Vet Res. 2019 Jul 29;15:263. doi: 10.1186/s12917-019-2006-6 (PMC6661835; doi:10.1186/s12917-019-2006-6)
Supplement: Supplementary file 1 — Table S1A. ANCOVA Model 1A: Bile-Extracted and Farm-Not-Extracted data adjusting for sex, season, and age (log transformed). The Difference is the estimated average difference in serum parameter (dependent variable) between the bear groups specified in the “Level” column. Table S1B. ANCOVA Model 1A: The difference in the effect of age on log Total White Blood Cell (TWBC). The “Estimate/Difference” is the estimated difference in the effect of age on TWBC between the bear groups specified in the “Level” column (log transformed). Table S2. ANCOVA Model 1B: Bile-Extracted and Farm-Not-Extracted data adjusting for sex and season (log transformed). Additional file tables provided for additional reference to results from the Analysis of Covariance (ANCOVA) models performed to assess for differences in serum biochemical and hematological parameters when accounting for season, sex, and/or age between BE and non-farmed, non-extracted bear groups. Estimated differences between categorical levels, standard errors, 95% confidence intervals, and p-values from the Analysis of Covariance (ANCOVA) models are presented in Tables 3, 4 and 5, and Table S1 and S2. A comparison of the effect of age between bear groups for TWBC is shown in Table 4 and Table S1B. (DOCX 24 kb) [file 12917_2019_2006_MOESM1_ESM.docx]

**Table S1A.** ANCOVA Model 1A: Bile-Extracted and Farm-Not-Extracted data adjusting for sex, season, and age (log transformed). The Difference is the estimated average difference in serum parameter (dependent variable) between the bear groups specified in the “Level” column.

| Serum Parameters | Obs | Bear Group  (*p*-value) | Interactions | | | Level | Difference | SE | Lower CI | Upper CI | *p-*value |
| --- | --- | --- | --- | --- | --- | --- | --- | --- | --- | --- | --- |
|  |  |  | Sex | Season | Age |  |  |  |  |  |  |
| ALT | 229 | 0.0018 | -- | -- | -- | BE-FNE | 0.488 | 0.154 | 0.184 | 0.793 | 0.002^a^ |
| GGT | 208 | <0.0001 | -- | -- | -- | BE-FNE | 1.073 | 0.222 | 0.635 | 1.510 | <0.0001^a^ |
| CREA | 219 |  | 0.0391 | -- | -- | F,BE-F,FNE  M,BE-M,FNE | -0.080  0.245 | 0.092  0.132 | -0.319  -0.096 | 0.160  0.586 | 0.8234^b^  0.2475^b^ |
| TP | 231 |  | 0.0455 | -- | -- | F,BE-F,FNE  M,BE-M,FNE | -0.0005  0.102 | 0.031  0.043 | -0.080  -0.009 | 0.079  0.213 | 1.000^b^  0.083^b^ |
| ALB | 231 |  | -- | 0.0201 | -- | Wi,BE-Wi,FNE  Su,BE-Su,FNE | 0.205  0.022 | 0.071  0.037 | 0.022  -0.074 | 0.389  0.119 | 0.022^b^  0.932^b^ |

Obs=number of observations; SE=standard error; CI=confidence interval; ALT=alanine aminotransferase; GGT=gamma glutamyl transferase; CREA=creatinine; TP=total protein; ALB=albumin; BE=bile-extracted; FNE=farm-not-extracted; F=female; M=male; Wi=winter season (December-April); Su=summer season (May-November); “- -“ indicates p-values > 0.05 and denotes interaction terms removed by stepwise selection, resulting in the reported significant p-values listed under Bear Group or Interaction columns after the other interactions terms were removed from the model; ^a^Student *t-*test, ^b^Tukey HSD

**Table S1B.** ANCOVA Model 1A: The difference in the effect of age on log Total White Blood Cell (TWBC). The “Estimate/Difference” is the estimated difference in the effect of age on TWBC between the bear groups specified in the “Level” column (log transformed).

| Model | Obs | Bear Group  (*p*-value) | Interactions | | | Level | Estimate/Difference | SE | Lower CI | Upper CI | *p-*value |
| --- | --- | --- | --- | --- | --- | --- | --- | --- | --- | --- | --- |
|  |  |  | Sex | Season | Age |  |  |  |  |  |  |
| 1A** | 227 |  | -- | -- | 0.0099 | BE-FNE | 0.0216 | 0.008 | 0.005 | 0.038 | 0.0099 |

“- -“ indicates p-values > 0.05 and denotes interaction terms removed by stepwise selection, resulting in the reported significant p-values listed under Interaction after the other interactions terms were removed from the model.

**Table S2.** ANCOVA Model 1B: Bile-Extracted and Farm-Not-Extracted data adjusting for sex and season (log transformed).

| Serum/Hematology  Parameters | Obs | Bear Group  (*p*-value) | Interactions | | Level | Difference | SE | Lower CI | Upper CI | *p-*value |
| --- | --- | --- | --- | --- | --- | --- | --- | --- | --- | --- |
|  |  |  | Sex | Season |  |  |  |  |  |  |
| ALT | 337 | <0.0001 | -- | -- | BE-FNE | .0309 | 0.078 | 0.155 | 0.464 | <0.0001^a^ |
| GGT | 298 |  | 0.0427 | -- | F,BE-F,FNE  M,BE-M,FNE | 0.471  0.975 | 0.171  0.184 | 0.029  0.500 | 0.912  1.449 | 0.0314^b^  <0.0001^b^ |
| LDH | 248 |  | -- | 0.0278 | Wi,BE-Wi,FNE  Su,BE-Su,FNE | -0.736  -0.390 | 0.122  0.097 | -1.052  -0.641 | -0.420  -0.138 | <0.0001^b^  0.0005^b^ |
| CK | 255 | <0.0001 | -- | -- | BE-FNE | 0.624 | 0.148 | 0.333 | 0.916 | <0.0001^a^ |
| CREA | 325 |  | <0.0001 | -- | F,BE-F,FNE  M,BE-M,FNE | -0.122  0.236 | 0.054  0.064 | -0.263  0.072 | 0.019  0.401 | 0.115^b^  0.0014^b^ |
| TP | 340 | <0.0001 | -- | -- | BE-FNE | 0.086 | 0.014 | 0.059 | 0.114 | <0.0001^a^ |
| ALB | 340 | <0.0001 | -- | -- | BE-FNE | 0.265 | 0.021 | 0.225 | 0.306 | <0.0001^a^ |
| GLOB | 339 | 0.0001 | -- | -- | BE-FNE | -0.110 | 0.028 | -0.166 | -0.055 | 0.0001^a^ |
| TWBC | 291 | <0.0001 | -- | -- | BE-FNE | 0.336 | 0.065 | 0.208 | 0.464 | <0.0001^a^ |

Obs=number of observations; SE=standard error; CI=confidence interval; ALT=alanine aminotransferase; GGT=gamma glutamyl transferase; LDH=lactate dehydrogenase; CK=creatine kinase; CREA=creatinine; TP=total protein; ALB=albumin; GLOB=globulin; TWBC=total white blood cell count; BE=bile-extracted; FNE=farm-not-extracted; F=female; M=male; Wi=winter season (December-April); Su=summer season (May-November); “- -“ indicates p-values > 0.05 and denotes interaction terms removed by stepwise selection, resulting in the reported significant p-values listed under Bear Group or Interaction columns after the other interactions terms were removed from the model; ^a^Student *t-*test, ^b^Tukey HSD
